# Comparison of AirSeal versus conventional insufflation system for retroperitoneal robot-assisted laparoscopic partial nephrectomy: a randomized controlled trial

**DOI:** 10.1007/s00345-024-04819-3

**Published:** 2024-02-21

**Authors:** Mengchao Wei, Wenjie Yang, Jingmin Zhou, Zixing Ye, Zhigang Ji, Jie Dong, Weifeng Xu

**Affiliations:** Department of Urology, Peking Union Medical College Hospital, Chinese Academy of Medical Sciences and Peking Union Medical College, Dongcheng, Beijing, 100000 China

**Keywords:** AirSeal, Insufflation system, Robotic surgery, Partial nephrectomy, Renal cell carcinoma

## Abstract

**Purpose:**

AirSeal is a valve-less trocar insufflation system which is widely used in robotic urologic surgeries. More evidence is needed concerning the application and cost of AirSeal in retroperitoneal robot-assisted laparoscopic partial nephrectomy.

**Methods:**

We conducted a randomized controlled trial enrolling 62 patients who underwent retroperitoneal robot-assisted laparoscopic partial nephrectomy from February 2022 to February 2023 in the Peking Union Medical College Hospital. Patients were randomly assigned into AirSeal insufflation (AIS) group and conventional insufflation (CIS) group. The primary outcome was the rate of subcutaneous emphysema (SCE).

**Results:**

The SCE rate in the AIS group (12.9%) was significantly lower than that in the CIS group (35.5%) (*P* = 0.038). Lower maximum end-tidal carbon dioxide (CO_2_) (41 vs 45 mmHg, *P* = 0.011), PaCO_2_ at the end of the operation (40 vs 45 mmHg, *P* < 0.001), maximum tidal volume (512 vs 570 ml, *P* = 0.003), frequency of lens cleaning (3 vs 5, *P* < 0.001), pain score at 8 h (3 vs 4, *P* = 0.025), 12 h (2 vs 3, *P* = 0.029) postoperatively and at time of discharge (1 vs 2, *P* = 0.002) were observed in the AIS group, despite a higher hospitalization cost (68,197 vs 64658RMB, *P* < 0.001). Logistic regression analysis identified insufflation approach was the only influencing factor for the occurrence of SCE events.

**Conclusion:**

AirSeal insufflation system exhibited similar efficacy and improved safety for retroperitoneal robot-assisted laparoscopic partial nephrectomy than conventional insufflation system, despite an affordable increase of hospitalization costs.

## Introduction

The incidence of renal cell carcinoma (RCC) is increasing, with approximately 400,000 new cases per year worldwide [1]. Partial nephrectomy is recommended for RCC whenever surgically applicable according to the latest European Association of Urology guideline [2]. Robot-assisted laparoscopic partial nephrectomy has been the standard approach to partial nephrectomy [3, 4] and shown an advantage over laparoscopic and open partial nephrectomy [5, 6].

For robot-assisted laparoscopic partial nephrectomy, a stable pneumocavity is critical. Conventional insufflation system utilized a one-way valve trocar to place instruments into the pneumocavity while maintaining insufflation [7]. However, a significant loss of insufflation may occur when passing instruments through the trocar or sucking smoke. Meanwhile, continuous insufflation may result in pressure spikes since the conventional insufflation system is closed [8]. Unstable pneumoperitoneum can cause the collapse of pneumocavity and moisture accumulation at the camera lens, thus disrupting the exposure of surgical field, prolonging operation time and increasing the risk of accidental injury. Furtherly, prolonged operation time can lead to excess absorption of carbon dioxide (CO_2_) which contributes to insufflation-related complications including subcutaneous emphysema (SCE) and shoulder pain [9, 10].

AirSeal is a three-lumen trocar insufflation system which creates a valve-less pressure curtain by continuous pressure flow [11]. The system can respond to the slight change of intra-abdominal pressure and create stable insufflation. Previous studies have shown that the system can improve visualization of the surgical field, enable continuous smoke sucking and reduce CO_2_ absorption and consumption [12, 13]. In the field of urologic surgery, AirSeal system has exhibited superiority over conventional insufflation system in robot-assisted laparoscopic nephrectomy [8, 14–16], prostatectomy [17, 18] and cystectomy [19].

Previous studies have fully investigated the efficacy and safety of AirSeal insufflation system in robot-assisted laparoscopic partial nephrectomy. However, special attention should be paid on retroperitoneal robot-assisted laparoscopic partial nephrectomy since the retroperitoneal cavity is more confined compared to transperitoneal cavity. Besides, there has been no evidence on the cost of AirSeal system in robotic urologic surgery.

Therefore, to shed further light upon the application of AirSeal in robot-assisted laparoscopic partial nephrectomy, we conducted a single-center, randomized controlled trial comparing the efficacy, safety and cost of AirSeal versus conventional insufflation system in robot-assisted laparoscopic partial nephrectomy via retroperitoneal approach.

## Materials and methods

The study was centrally approved by the Ethics Committee in accordance with the Declaration of Helsinki. The Approval Number was I-22PJ903.

### Patients

We prospectively enrolled patients who underwent retroperitoneal robot-assisted laparoscopic partial nephrectomy from February 2022 to February 2023 in the Peking Union Medical College Hospital. The inclusion criteria were as follows: (1) patients were aged between 18 and 80 years; (2) patients were diagnosed as a single renal lesion with a size within 6 cm; (3) patients were planned to undergo retroperitoneal robot-assisted laparoscopic partial nephrectomy; (4) patients were capable to give informed consent. Patients with active systemic or cutaneous infection, pre-existing immunodeficiency disorder and/or chronic use of systemic steroids, uncontrolled diabetes mellitus, ascites, body mass index (BMI) greater than 45 kg/m^2^ or less than 18 kg/m^2^, severe co-existing morbidities, significant anaemia with haemoglobin (Hb) less than 10 g/dl, renal insufficiency with creatine (CREA) greater than 2.5 mg/dl, and significant history of bleeding diathesis, coagulopathy or von Willebrand’s disease were excluded from enrolment. Females who were pregnant or planning to become pregnant within 3 months of surgery, or lactating were also excluded.

### Randomization and masking

Patients were randomly assigned in a 1:1 ratio into AirSeal insufflation (AIS) group and conventional insufflation (CIS) group. Randomization was performed by an online random number generator (www.random.org) with clinical team and research staffs masked. Though the surgical procedure could not be masked due to different insufflator appearances, the pathological diagnosis, inpatient care, outpatient follow-up and statistical analysis were all masked.

### Data collection and outcomes

Patients’ clinical characteristics including sex, age, BMI, history of smoking, hypertension and diabetes, Charlson comorbidity index (CCI), side and size of the tumour lesion, and RENAL score were collected.

The primary outcome was the rate of SCE assessed by the surgeon at the end of the operation. SCE was categorized as “clinically significant” (as determined by measurement at neck or head level), “subclinical” (only seen around port sites or to chest level), or not present [15]. Secondary outcomes included operation time (from the time when the pneumoperitoneum was established to the time when the pneumoperitoneum was finished), warm ischemia time, blood loss, maximum peak airway pressure, maximum end-tidal CO_2_, PaCO_2_ at the end of the operation, maximum tidal volume, the frequency of lens cleaning, perioperative Hb difference, perioperative CREA difference, pain score evaluated by visual analog scale and obtained at 4, 8, 12 h postoperatively and at time of discharge, postoperative hospital stay and hospitalization costs. Peak airway pressure, end-tidal CO_2_ and tidal volume were measured every 15 min during the surgery.

### Operations

The da Vinci Xi surgical robot system was used for all patients. The standard pressure was set as 12 mmHg in the AIS group and 15 mmHg in the CIS group. A retroperitoneal 4-port partial nephrectomy was performed in both groups. The AirSeal system is composed of an Intelligent Flow System control unit, one valve-less access port and one contiguous trilumen filter tube set. In the AIS group, a 12-mm AirSeal trocar was placed as the assistant port instead of a regular trocar. All the operations were performed by the same team of surgeon and assistant with a 10-year experience. The patient positioning, port placement, robotic instrument use and detailed surgical procedures were the same as described in our previously published research [20].

### Statistical analysis

The sample size calculation was based on the primary outcome of SCE. The one-sided type I error rate was set at 10% and type II error rate set at 20%, giving 80% power. Based upon the previous study [15], we hypothesized that the SCE rate was 38.5% in the CIS group and 15.2% in the AIS group. It was estimated that 62 patients were required. Following randomizing the sample size in a 1:1 ratio, 31 patients were needed in each group.

Continuous variables were presented as median and interquartile range (IQR). Categorical variables were presented as frequency and percentage. Difference test of the primary outcome was conducted using the chi-square test. The secondary outcomes were presented as median and IQR and compared using Mann–Whitney *U* test. Logistic regression analysis was used to determine factors influencing SCE. Statistical analyses were conducted using SPSS software (version 25, IBM). All tests were two-sided, and *P* < 0.05 indicated statistical significance.

## Results

### Patient characteristics

**Fig. 1:**
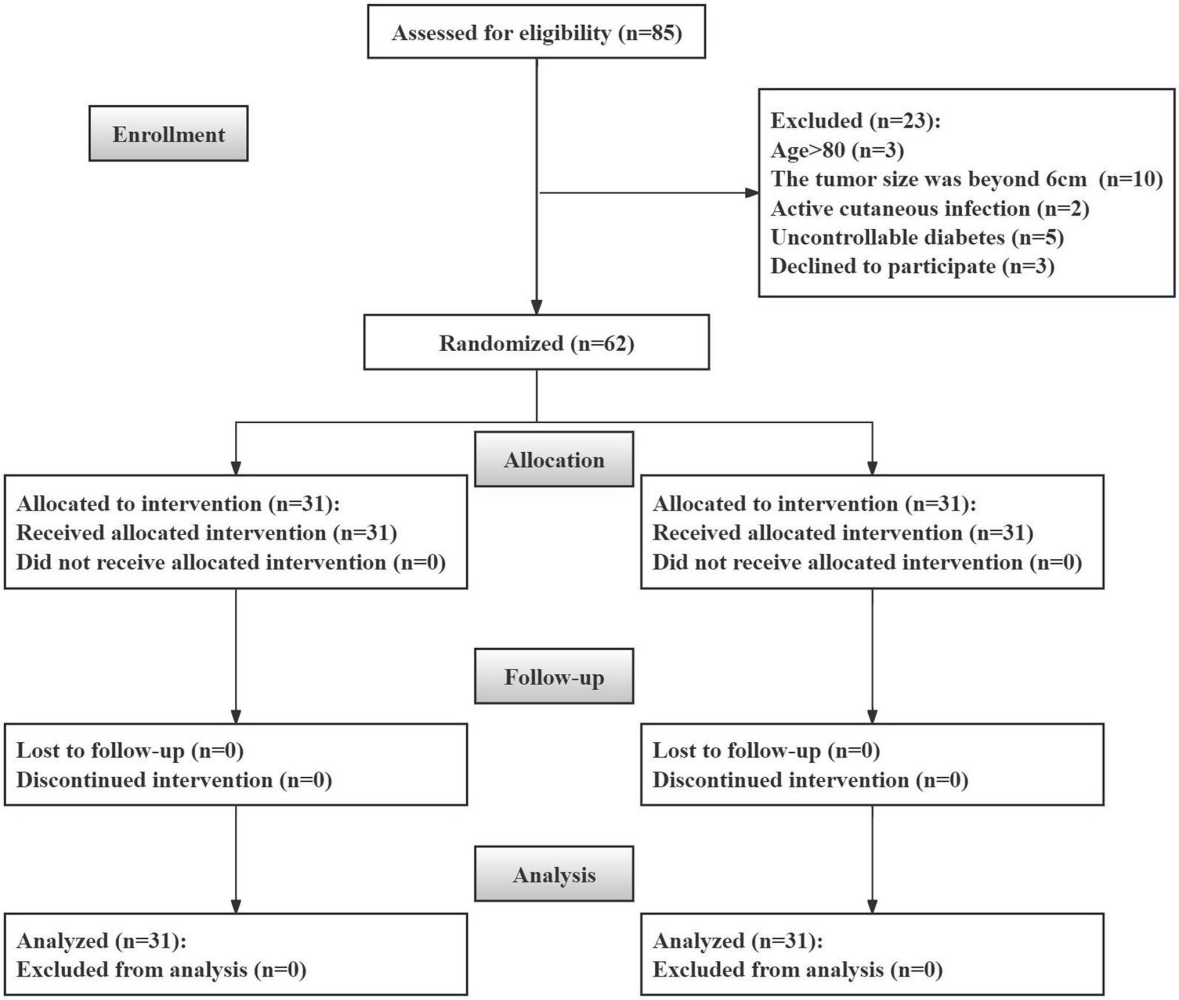
The flow chart of the study

A total of 62 patients were eventually enrolled in the study, with 31 patients in each group. The flow chart of randomization was shown in Fig. 1. Baseline characteristics of the two groups were shown in Table 1. All characteristics were comparable between the two groups. The median age of the whole cohort was 53.0 years (IQR: 46.8–63.3 years). There were 37 men and 25 women. The median tumour size was 3.2 cm (IQR: 2.2–4.3 cm). The median RENAL score was 7 (IQR: 5.8–9). Pathologic analysis showed that all the renal lesions were RCC. All the operations were performed successfully without conversion to open surgery.

**Table 1:** Baseline characteristics of the CIS group and AIS group

Characteristics	CIS (*n* = 31)	AIS (*n* = 31)
Sex (man/woman)	17(54.8)/14(45.2)	20(64.5)/11(35.5)
Age, years	57(44, 63)	53(48, 64)
BMI, kg/m^2^	24.3(22.6, 27.0)	25.4(22.7, 27.7)
Smoking (yes/no)	8(25.8)/23(74.2)	10(32.3)/21(67.7)
Hypertension (yes/no)	18(58.1)/13(41.9)	14(45.2)/17(54.8)
Diabetes (yes/no)	7(22.6)/24(77.4)	5(16.1)/26(83.9)
CCI	3(2, 5)	3(2, 4)
Tumor side (left/right)	16(51.6)/15(48.4)	15(48.4)/16(51.6)
Tumor size, cm	2.9(2.0, 3.8)	3.5(2.5, 4.5)
RENAL score	7(5, 9)	7(6, 9)

*CIS* conventional insufflation, *AIS* AirSeal insufflation, *BMI* body mass index, *CCI* Charlson comorbidity index

### Outcomes

The incidence rate of SCE, including clinically significant SCE and subclinical SCE, was significantly lower in the AIS group than the CIS group (12.9% vs 35.5%, *P* = 0.038). Nearly all the SCE events were subclinical but in the CIS group, two patients developed clinically significant SCE extending to neck. They had local discomfort without dyspnoea, distress and chest pain and recovered within one week without surgical or medical intervention. The outcomes were summarized in Table 2. Patients in the AIS group had significantly lower maximum end-tidal CO_2_ (41 vs 45 mmHg, *P* = 0.011), PaCO_2_ at the end of the operation (40 vs 45 mmHg, *P* < 0.001), maximum tidal volume (512 vs 570 ml, *P* = 0.003), frequency of lens cleaning (3 vs 5, *P* < 0.001), pain score at 8 h (3 vs 4, *P* = 0.025), 12 h (2 vs 3, *P* = 0.029) postoperatively and at time of discharge (1 vs 2, *P* = 0.002). However, a higher hospitalization cost was observed in the AIS group (68,197 vs 64,658RMB, *P* < 0.001). Operation time, warm ischemia time, blood loss, maximum peak airway pressure, perioperative Hb difference, perioperative CREA difference, pain score at 4 h and postoperative hospital stay were comparable between the two groups.

**Table 2:** Outcomes of the CIS group and AIS group

Outcomes	CIS (*n* = 31)	AIS (*n* = 31)	*P* value
Subcutaneous emphysema (yes/no)	11(35.5%)/20(64.5%)	4(12.9%)/27(87.1%)	0.038*
Operation time, min	100(90, 130)	90(90, 128)	0.704
Warm ischemia time, min	20(15, 25)	19(15, 24)	0.810
Blood loss, ml	50(20, 50)	50(20, 50)	0.341
Maximum peak airway pressure, mmHg	24(23, 28)	23(20, 25)	0.118
Maximum end-tidal CO_2_, mmHg	45(40, 47)	41(36, 44)	0.011*
PaCO_2_ at the end of the operation, mmHg	45(41, 53)	40(35, 41)	<0.001*
Maximum tidal volume, ml	570(505, 644)	512(478, 557)	0.003*
Frequency of lens cleaning	5(5, 5)	3(3, 3)	<0.001*
Perioperative Hb difference, g/L	17(10, 26)	15(10, 23)	0.647
Perioperative CREA difference, μmol/L	15(9, 23)	14(5, 29)	0.485
Pain score at 4h	5(3, 8)	4(3, 6)	0.144
Pain score at 8h	4(3, 7)	3(3, 4)	0.025*
Pain score at 12h	3(2, 5)	2(2, 4)	0.029*
Pain score at time of discharge	2(1, 3)	1(1, 2)	0.002*
Postoperative hospital stay, day	5(4, 6)	5(4, 6)	0.959
Hospitalization costs, RMB	64658(62636, 69181)	68197(66162, 70543)	<0.001*

*CIS* conventional insufflation, *AIS* AirSeal insufflation, *CO*_2_ carbon dioxide, *Hb* hemoglobin, *CREA* creatine

*Statistically significant at *α* = 0.05

### Factors influencing the occurrence of SCE

As shown in Table 3, univariable logistic regression analysis revealed that insufflation approach was the only influencing factor for the occurrence of SCE events (OR = 4.263; 95% CI 1.192–15.252; *P* = 0.026).

**Table 3:** Logistic regression analysis for factors influencing SCE

Characteristics	Univariable analysis		
	*β*	OR (95% CI)	*P* value
Sex (man)	−0.191	0.827 (0.261, 2.615)	0.746
Age, years	−0.010	0.990 (0.945, 1.036)	0.658
BMI, kg/m^2^	0.183	1.201 (0.979, 1.474)	0.080
Smoking (no)	−0.531	0.588 (0.176, 1.967)	0.389
Hypertension (no)	0.087	1.091 (0.350, 3.404)	0.881
Diabetes (no)	1.551	4.714 (0.558, 39.853)	0.155
CCI	−0.123	0.885 (0.608, 1.287)	0.522
Tumor side (left)	−0.338	0.713 (0.227, 2.240)	0.562
Tumor size, cm	0.122	1.130 (0.720, 1.775)	0.595
RENAL score	0.096	1.101 (0.805, 1.507)	0.548
Insufflation approach (CIS)	1.450	4.263 (1.192, 15.252)	0.026*

*SCE* subcutaneous emphysema, *OR* odds ratio, *CI* confidence interval, *BMI* body mass index, *CCI* Charlson comorbidity index, *CIS* conventional insufflation system

*Statistically significant at *α* = 0.05.

## Discussion

To further illustrate the role of the application of AirSeal in robot-assisted laparoscopic partial nephrectomy, we prospectively enrolled 62 RCC patients who underwent retroperitoneal robot-assisted laparoscopic partial nephrectomy using conventional or AirSeal insufflation system. Results revealed that AirSeal insufflation system exhibited less SCE rate, end-tidal CO_2_, PaCO_2_, tidal volume, frequency of lens cleaning, and postoperative pain compared to conventional insufflation system, despite a higher hospitalization cost.

Retroperitoneal approach is widely used in robot-assisted laparoscopic partial nephrectomy [21]. Compared to transperitoneal approach, the space of retroperitoneal cavity is smaller and less distensible [22]. Therefore, a stable pneumoperitoneum is more essential for a better exposure of the surgical field and the smooth running of the operation. AirSeal insufflation system can ensure a stable pneumoperitoneum with sustaining smoke evacuation during surgery, providing a useful tool for robotic retroperitoneal partial nephrectomy. However, despite the less frequency of lens cleaning in the AIS group, our study found no significant differences in operation time, warm ischemia time and blood loss between the AIS and CIS groups. The rich experience of the surgical team and the relatively small size and common complexity of the tumour lesions may explain this.

Besides, given the lack of the peritoneum lining, the inflated air can spread within the retroperitoneal tissues and furtherly into loose subcutaneous layer. Therefore, retroperitoneal approach can cause a higher SCE rate than transperitoneal approach [15]. This disadvantage may be overcome to some extent since AirSeal can allow a lower pre-set pneumoperitoneum pressure and reduce the occurrence of pressure spikes. Our study reported a SCE rate of 12.9% in the AIS group, similar to 15.2% in the previous study [15], significantly lower than 35.5% in the CIS group. Moreover, insufflation approach was an independent influencing factor for the occurrence of SCE events.

In terms of CO_2_ pressure, unstable pneumoperitoneum can cause increased CO_2_ absorption and higher end-tidal CO_2_ and PaCO_2_ which are associated with reduced venous flow and respiratory compliance [23]. More tidal volume is needed to expel excess CO_2_. In addition, a higher CO_2_ pressure may contribute to overstretching of the diaphragmatic muscle fibres and postoperative shoulder pain [9]. Our study observed less end-tidal CO_2_, PaCO_2_, tidal volume and postoperative pain in the AIS group, indicating the advantage of AirSeal insufflation system in reducing CO_2_ absorption.

Until now, there have been three major studies investigating the efficacy and safety of AirSeal insufflation system for robot-assisted laparoscopic partial nephrectomy [14–16], including two randomized controlled trials and one prospective cohort study. Filippo Annino et al. firstly compared the AirSeal with a standard insufflator system and found that patients in the AirSeal group had shorter operative time and warm ischemia time [14]. A large prospective randomized clinical trial demonstrated that 12 mmHg AirSeal insufflation improved intraoperative cardiopulmonary parameters and safety profile compared to 15 mmHg AirSeal insufflation and conventional insufflation in robot-assisted laparoscopic partial nephrectomy [15]. Feng et al. also noted that 12 mmHg AirSeal insufflation was associated with reduced risk of SCE and shoulder pain in robot-assisted laparoscopic partial nephrectomy [16]. Besides, retroperitoneal surgical approach was a significant predictor for developing SCE, possibly due to ease of CO_2_ tracking without barriers as peritoneum in transperitoneal approach. In comparison with previous studies, we first adopted PaCO_2_ as a secondary outcome. PaCO_2_ is usually measured via extraction of arterial blood and can reflect a more precise level of intracorporal CO_2_ than end-tidal CO_2_ [24]_._ Besides, we firstly investigated the cost of AirSeal insufflation system. A difference of 3539RMB (about 5% of the total hospitalization costs) in hospitalization costs between AIS and CIS groups was observed, which was affordable for the majority of Chinese patients.

Our current study has several strengths. Unlike previous studies evaluating AirSeal insufflation system for robot-assisted laparoscopic partial nephrectomy, we focused on patients undergoing retroperitoneal approach. Besides, as mentioned above, we first adopted PaCO_2_ and hospitalization costs as secondary outcomes, providing more insights for the role of AirSeal insufflation system. However, there are several limitations in our study as well. First, the sample size of our study was relatively small. Large randomized controlled trials are needed to provide more solid evidence on this issue. Second, our result for hospitalization costs is only suitable for Chinese patients. For patients from other regions, the cost of AirSeal should be re-evaluated. Third, the median tumour size was only 3.2 cm and the median RENAL score was only 7, reflecting a relatively medium difficulty of partial nephrectomy in our study. Besides, the surgical team had a 10-year surgical experience. Whether AirSeal insufflation system is efficacious and safe for more challenging RCC and less experienced surgical teams is to be determined.

In summary, our randomized controlled trial comparing AirSeal against conventional insufflation system demonstrated similar efficacy and improved safety of AirSeal insufflation system for retroperitoneal robot-assisted laparoscopic partial nephrectomy, despite an affordable increase of hospitalization costs.

## Data Availability

The data that support the findings of this study are available from the corresponding author upon reasonable request.
